# Initial follow‐up of mechanical QC tests for RapidArc Dynamic (RAD)

**DOI:** 10.1002/acm2.70411

**Published:** 2025-12-28

**Authors:** Emma Van Riet, Wout Piot, Bianca Vanstraelen, Karel Aerts, Truus Reynders, Tom Depuydt, Wouter Crijns

**Affiliations:** ^1^ Laboratory of Experimental Radiotherapy Department of Oncology KU Leuven Leuven Belgium; ^2^ Department of Radiation Oncology University Hospitals UZ Leuven Leuven Belgium

**Keywords:** dynamic collimator, mechanical QC, picket fence, RapidArc Dynamic (RAD)

## Abstract

**Background:**

RapidArc Dynamic (RAD) is a new solution for delivery of radiotherapy introduced by Varian. RAD blends Volumetric Modulated Arc Therapy (VMAT) and Intensity‐Modulated Radiation Therapy (IMRT) by pausing gantry rotation at predefined positions and adds a degree of freedom: a dynamic collimator.

**Purpose:**

Two new tests validate the dynamic collimator's impact during RAD. The UZ Leuven developed picket fence test verifies MLC movement in combination with a rotating collimator. The Pac‐Man test (designed by Varian) verifies the synchronous working of various collimator speeds and dose rates.

**Methods:**

The RAD picket fence test was performed using radiochromic film. For the analysis, the measured distances between the pickets and a reference jaw were compared to the actual distances. To validate the results, the test was also performed for RapidArc. For the Pac‐Man test, acquired using an Electronic Portal Imaging Device (EPID), five regions of interest (ROIs) with varying dose rates and collimator speeds were analyzed. All ROIs were normalized with an open field image, and the percentage deviation against the average of all ROIs (Diff(x)), as well as the absolute average of all differences ($\mathrm{Dif}f_{\mathrm{Abs}}$) was calculated. To validate, the Pac‐Man test was also executed using film. Both tests were conducted over 12 weeks and results were compared to thresholds found in literature (AAPM TG‐142, MPPG 8.b). To verify stability, control limits were calculated based on AAPM TG‐218.

**Results:**

The picket fence test results showed a maximum difference of 0.45 mm (±0.05) and a mean difference of 0.14 mm (± 0.01). For the Pac‐Man test, the Diff(x) values ranged between –1.31% and 1.02% and the $\mathrm{Dif}f_{\mathrm{Abs}}$ value between 0.42% and 0.55%. All results fell within the calculated control limits, indicating stability.

**Conclusions:**

These consistent results, which fall under thresholds set in literature, indicate that RAD plan delivery is mechanically feasible. The two introduced tests will from now on be included in the routine mechanical QC in UZ Leuven.

## 1 INTRODUCTION

RapidArc Dynamic (RAD), created by Varian Medical Systems, is a recent innovation for planning and delivery of radiotherapy on Eclipse and TrueBeam systems. It combines the benefits of both Intensity‐Modulated Radiation Therapy^1^ (IMRT) and Volumetric Modulated Arc Therapy (VMAT)^2^ while adding a new degree of freedom: a rotating dynamic collimator. During treatment, VMAT gantry rotation can be strategically paused at selected angles to deliver up to 10 static IMRT fields. Furthermore, a dynamic collimator is implemented, allowing for optimal dose delivery.

VMAT offers reduced treatment time and increased efficiency and is mostly used for indications such as head and neck cancer, prostate cancer, and so on. IMRT allows for precise directional control and is frequently used in anatomies where one would choose to avoid a low‐dose bath, such as in lung and esophageal cancer.^3^ Efforts to combine IMRT and VMAT, e.g., in hybrid planning for breast cancer,^4^, ^5^ esophageal cancer,^6^ and unresectable olfactory neuroblastoma,^7^ have shown improved target conformity, as well as reduced dose to the neighboring organs at risk. Adding a dynamic collimator to this IMRT‐VMAT hybridization will further enhance the dose distribution.

As with the commissioning of any radiotherapy technique, mechanical validation is essential. Dynamic delivery techniques such as VMAT, IMRT, and RAD require precise synchronization of dynamic machine parameters. For IMRT and VMAT, established QC measures include a picket fence test (for IMRT^8^ and VMAT^9^), the dose rate gantry speed test (DRGS, for VMAT^9^), and the dose rate MLC leaf speed test (DRMLC, for VMAT^9^). These tests cover the synchronization of key dynamic parameters, namely dose rate, multileaf collimator (MLC) leaf speed, and gantry rotation. However, with the introduction of a rotating dynamic collimator in RAD, this added degree of freedom also requires mechanical validation. Delivery‐timing errors, such as misalignment between leaf motion and collimator rotation, could lead to clinically significant deviations that may not be fully captured by patient‐specific QA. The regular execution of synchronization tests is thus essential to verify dynamic control performance. Therefore, two new mechanical QC tests are introduced: The ‘Pac‐Man’ test^10^, designed by Varian and more officially called the dose rate collimator speed test (DRCS), assesses collimator and dose rate modulation, combined with a rotating gantry. During this test, the MLCs remain static, so another test was required to validate the synchronous operation of the MLC movement, rotating gantry, and rotating collimator: a RAD picket fence test. This supplementary collimator rotation results in the outermost 5 mm leaves having an additional linear velocity of 1 cm/s, while the outermost 10 mm leaves have an additional linear velocity of 2 cm/s—a new mechanical feature that requires follow‐up.

This study evaluated the mechanical feasibility of a TrueBeam Linac configured for RAD delivery (the first clinically implemented system of its kind) using these two dedicated tests over a period of 12 weeks. To validate the RAD picket fence test, the test was also carried out for RapidArc (RA; collimator‐static) for 8 days. This was done to confirm that possible higher inaccuracies are due to the test method (film) and not the rotating collimator. For validation of the Pac‐Man test, to ensure that the variations found in the results were due to the machine performance and not the test method (Electronic Portal Imaging Device; EPID), the test was once carried out using radiochromic film.

## 2 MATERIALS AND METHODS

All tests were performed on a TrueBeam 4.1 (Millenium MLC) Linac. Dedicated DICOM plans were created for each test (Varian‐provided for the Pac‐Man test) and delivered in clinical mode.

### 2.1 Picket fence test

**FIGURE 1:**
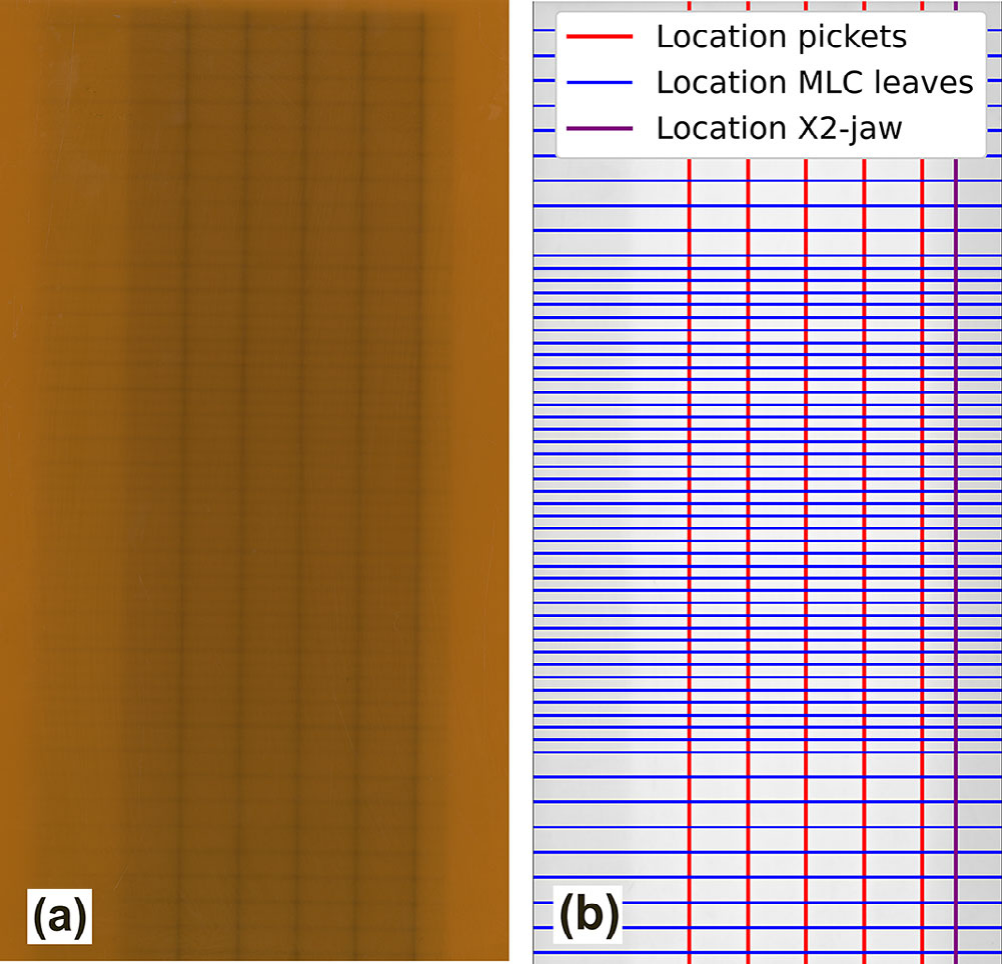
(a) Scanned picket fence test film and (b) overview of the analysis.

The in‐house developed RAD picket fence test resembles a conventional RA picket fence test in terms of leaf and gantry motion but integrates dynamic collimator rotation. For the first picket, both the gantry and collimator remain static. For the subsequent four pickets (totaling five), the gantry and collimator move at a constant speed of 5.75°/s. A fixed dose rate of 1000 MU/min is used for the whole test. The pattern (Figure 1a) is measured using radiochromic film (Gafchromic RTQA2‐1010^11^) positioned on a film holder, which is inserted into the Linac accessory mount attached to the gantry head, resulting in a Source‐to‐Film Distance (SFD) of 65.2 cm. The exposed films are scanned using a 16‐bit EPSON‐GT‐15000 Flatbed Scanner at a resolution of 300 dpi and analyzed for MLC positioning accuracy (Figure 1b). Each picket location is defined using peak detection for each MLC leaf pair, after which the absolute distance (in mm; 300 dpi ∼ 0.085 mm/pixel) to the X2‐jaw is calculated. The X2‐jaw serves as reference due to its position remaining constant during the test (positional stability of 0.1 mm^12^). Since the film is located at an SFD of 65.2 cm, a correction factor of 100/65.2 is applied to project the measured distances to SAD 100 cm to compare them to the actual distances as obtained from the RT‐Plan. The test was carried out 17 times: daily for 7 days after implementation and once a week thereafter, totaling 12 weeks. An energy of 6 MV was used for the test.

To validate the results, the picket fence test was also performed for RA on 8 days. This was implemented by using the same plan and disabling the collimator rotation. To minimize uncertainty, the RAD and RA picket fence tests were carried out consecutively. Normality of the datasets was assessed with the Shapiro‐Wilk test, followed by either a paired *t*‐test or a Wilcoxon signed‐rank test for statistical comparison.

### 2.2 Pac‐Man test

**FIGURE 2:**
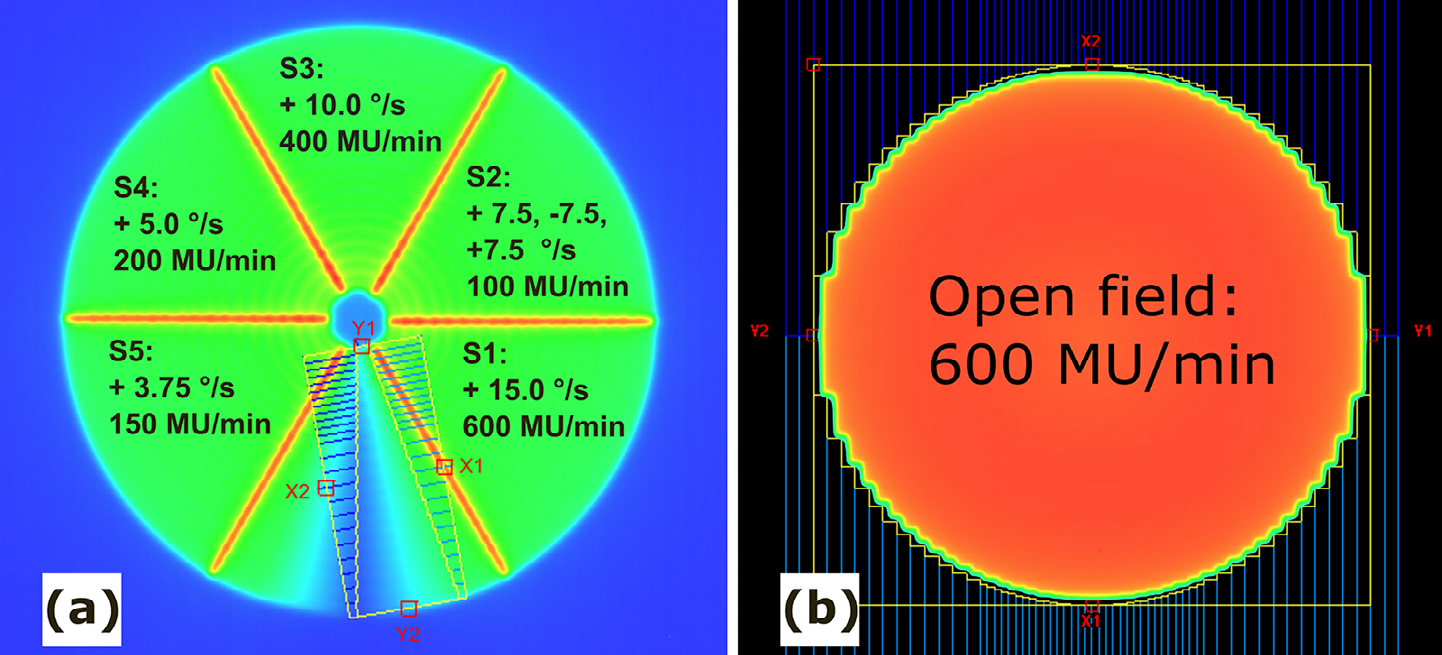
(a) Overview of the five different segments with varying collimator speed (°/s) and dose rate (MU/min) in the Pac‐Man test, showing the static MLC V‐shape used to create the segments. (b) The normalization field and the MLC pattern used to create the field.

The Varian‐provided Pac‐Man test examines five different combinations of collimator speed and dose rate across five gantry sub‐arcs, resulting in the same monitor units per sub‐arc. During each sub‐arc, the MLC remains in a static V shape while the collimator and the gantry rotate to create the wedge‐shaped pattern from Figure 2a. The gantry travels at a constant rate of 6°/s (maximum gantry speed). Separating each sub‐arc is a 2 mm MLC static gap delivered at a dose rate of 600 MU/min, with collimator and gantry held stationary. The pattern itself is measured using EPID. To eliminate machine output effects (non‐flatness/asymmetry), both a dynamic and a normalization field are required. For normalization, one static, open, circular field (Figure 2b) is used, delivered at 600 MU/min at a gantry angle of 181° and with a static collimator. For the analysis, five wedge‐shaped regions of interest (ROI) are selected within the five segments of both the open‐field and the RAD images. For each ROI, the following parameters are calculated:

‐ The mean pixel value of each ROI in the Pac‐Man field (*R*_PM_) corrected against the mean pixel value of the corresponding ROI in the open field (*R*_O_):
$$\circ \;R_{\mathrm{corr}}\;\left(x\right)=\frac{R_{\mathrm{PM}}\;\left(x\right)}{R_{O}\;\left(x\right)}\cdot 100$$
‐ The percentage deviation of each ROI *R*_corr_(x) calculated against the average $\bar{R_{\mathrm{corr}}\left(x\right)}$ of all ROIs:
$$\circ \;\mathrm{Diff}\;\left(x\right)=\frac{R_{\mathrm{corr}}\;\left(x\right)}{\bar{R_{\mathrm{corr}(x)}}}\cdot 100-100$$
‐ Average of absolute deviations across ROIs:
$$\circ \;\mathrm{Dif}f_{\mathrm{Abs}}=\bar{\left|\mathrm{Diff}(x)\right|}$$

The test was performed 20 times for each of the three energies (6 MV, 10 MV, and 6 MV‐FFF); daily during the first week of implementation and once or twice a week for the following 11 weeks, totaling 12 weeks. To ensure that the variations found in the results were due to the machine performance and not the EPID, the test was once carried out using 6 MV with a radiochromic film (Gafchromic RTQA2‐1010) pasted on the EPID.

All analyses (for both tests) were performed with an in‐house Python script (version 3.12). To verify the consistency of the data, control limits were calculated based on AAPM TG‐218^13^: $\mathrm{CL}=\mathrm{center}\;\mathrm{line}\pm 2.66\times \bar{mR}$ where centerline is the average of all measurements and $\bar{mR}$ is the average moving range.

## 3 RESULTS

### 3.1 Picket fence test

**FIGURE 3:**
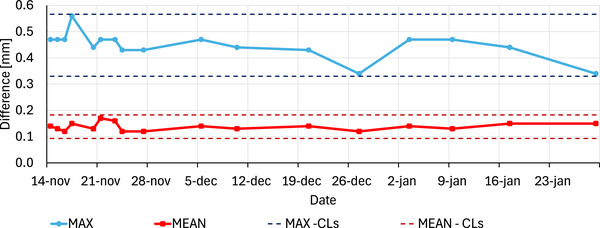
Trendlines of maximum and mean differences (mm) between measured and actual picket fence positions across all MLC leaf pairs, with control limits (CLs) indicated.

For the maximum and mean differences, values of 0.45 mm (±0.05) and 0.14 mm (±0.01) were obtained. The results were also very consistent over time, as shown in Figure 3. For the maximum difference, the lower and upper control limits were 0.33 and 0.57 mm and for the mean difference, they were 0.09 and 0.18 mm. All test results fell within these calculated control limits.

Regarding the comparison between RAD and RA picket fence tests, no statistically significant difference was found between the data, as shown in Table 1.

**TABLE 1:** Maximum and mean differences (mm) between measured and actual picket positions for RapidArc Dynamic (RAD) and RA (*n* = 8).

	Max. difference (mm)	Mean difference (mm)
RAD	0.43 ± 0.05	0.14 ± 0.01
RA	0.46 ± 0.06	0.15 ± 0.01
*p*‐Value	0.19^+^	0.34^++^

*Note*: Statistical comparison used Wilcoxon signed rank test (+) or paired‐*t* test (++), based on data normality.

### 3.2 Pac‐Man test

**FIGURE 4:**
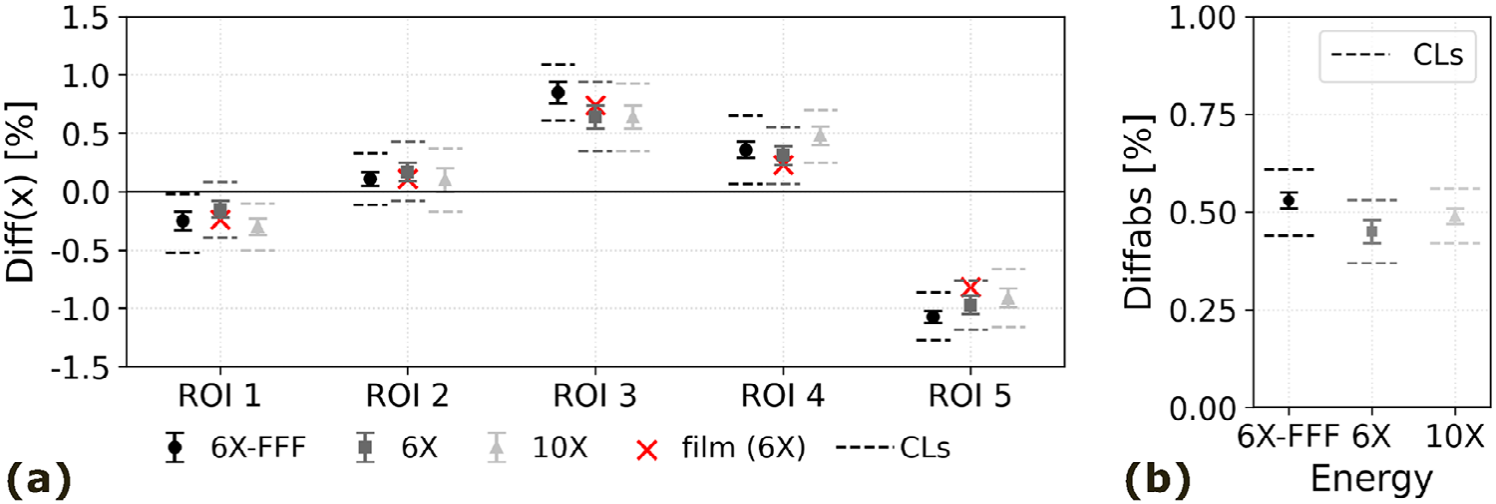
Pac‐Man test results (Diff(*x*) (a) and Diff_Abs_ (b)) for three energies (6X‐FFF, 6X, 10X). Mean values and standard deviations are indicated based on data from 20 measurements. The red crosses indicate the film measurement results. The control limits (CLs) are indicated with dashed lines.

The results for the Diff(x) and $\mathrm{Dif}f_{\mathrm{Abs}}$ values can be found in Figure 4. The Diff(x) values ranged between −1.31% and 1.02% and the $D\mathrm{if}f_{\mathrm{Abs}}$ value between 0.42% and 0.55% for the three different energies. For all three energies, the control limits were calculated and tabulated in Table 2. All measured results for all three energies fell within these control limits. As for the comparison between EPID and film measurements, the results can also be found in Figure 4.

**TABLE 2:** Control limits (CLs) (%) for Diff(*x*) and Diff_Abs_ values for all three energies (6X, 10X, and 6X‐FFF).

	Diff(x)					
CLs (%)	ROI 1	ROI 2	ROI 3	ROI 4	ROI 5	$\mathrm{Dif}f_{\mathrm{Abs}}$
6X	[−0.39, 0.08]	[−0.08, 0.43]	[0.35, 0.94]	[0.07, 0.55]	[−1.18, −0.76]	[0.37, 0.53]
10X	[−0.50, −0.10]	[−0.17, 0.37]	[0.35, 0.93]	[0.25, 0.70]	[−1.16, −0.66]	[0.42, 0.56]
6FFF	[−0.52, −0.02]	[−0.11, 0.33]	[0.61, 1.09]	[0.07, 0.65]	[−1.27, −0.86]	[0.44, 0.61]

## 4 DISCUSSION

The RAD picket fence test demonstrated high mechanical accuracy in the delivery of RAD plans, with the maximum difference of 0.45 mm falling well within the 1 mm limit for MLC positional accuracy set by AAPM TG‐142.^14^ Furthermore, all measured results were within the calculated control limits, indicating process stability and consistency over the evaluated period.

The latest AAPM guidance on MLC positional QC, as outlined in MPPG 8.b,^15^ tightens the action limit to 0.5 mm. While the results approach this threshold, it should be noted that radiochromic film was used instead of the standard EPID. The use of an EPID was not feasible here due to it being static with respect to the rotating collimator, which would distort the picket fence pattern. Unfortunately, using film reduces accuracy because of positioning inaccuracies. However, the observed inaccuracies likely stem from the film‐based method rather than the test itself, as confirmed by the non‐significant differences between RAD and RA. Therefore, a 1 mm action limit is implemented for the RAD picket fence test, with TG‐218 control limits adopted as tolerance limits.

While a comparison with a standard picket fence test at static gantry angles could provide additional insight into the effect of collimator rotation, AAPM MPPG 8.b^15^ recommends the same 0.5 mm action limit for both static and arc deliveries, and only ≤0.2 mm positional differences were observed between static‐gantry and RA picket fence films,^9^ suggesting that equivalent MLC positional accuracy can be expected across delivery types. Therefore, this test was not included in our study, but such a comparison may be of interest for future work.

All Pac‐Man test results fell within the thresholds set for the analytically corresponding DRGS and DRMLC speed tests^16^ (±3.0% for Diff(x) and 1.5% for $\mathrm{Dif}f_{\mathrm{Abs}}$). The results for the $\mathrm{Dif}f_{\mathrm{Abs}}$ value even remain under 0.55% for all three energies, indicating that the Linac is performing well within expected parameters when delivering RAD plans. For all three energies the results were consistent over time, which can be concluded from the narrow control limit range in which all results fell. These results indicate process stability for all three energies. Based on these findings, only the 6 MV Pac‐Man test will be used for routine QC, applying the calculated control limits as tolerance thresholds.

Minor energy dependence was observed, with 6 MV‐FFF showing the largest deviations. This may be attributed to the intrinsic beam characteristics of FFF delivery, such as less beam flatness, which can make the system more sensitive to minor timing or motion inaccuracies during dynamic delivery. Furthermore, when imaging FFF beams with an EPID, the phosphor screen shows an increased sensitivity to the low‐energy photons present in typical FFF beam spectra.^17^

Secondly, segment‐wise deviations showed a certain pattern: the largest positive deviation was found in segment 3, followed by segments 4 and 2. Segments 1 and 5 showed negative deviations compared to the average segment output. These deviations were confirmed by an independent film measurement, suggesting that they are inherent to the Linac and not the EPID. Possible causes include mechanical stress from high collimator speeds affecting motor timing or synchronization issues between gantry and collimator rotation. Future studies could explore this further using reversed gantry or collimator rotation or static gantry setups.

It should be noted that this study was conducted using a single TrueBeam machine. While the results demonstrate consistent performance and reliability within this setup, we acknowledge that broader validation across multiple machines would strengthen the generalizability of the findings.

Based on MPPG 8.b^15^ guidelines, which recommend frequent quantitative checks of both MLC leaf position accuracy and dynamic delivery control, we propose executing the RAD picket fence and Pac‐Man tests alongside the DRGS and DRMLC tests. The RAD picket fence test, which includes one collimator‐stationary picket, can replace the VMAT/IMRT picket fence test performed on a 2‐month frequency. This bi‐monthly schedule aligns with our QA framework and is supported by the stability observed over 12 weeks. Other centers may choose different frequencies based on their own QA framework, however.

In accordance with AAPM MPPG 8.b,^15^ QC results exceeding tolerance limits should trigger verification of measurement integrity and setup, along with systematic monitoring, while clinical operation may safely continue. If consistent trends are observed, a review of the mechanical performance should be initiated, and the tolerance limits may be refined or corrective actions implemented as appropriate. Major deviations, however, should trigger immediate mechanical verification of all relevant components, including vendor consultation.

## 5 CONCLUSION

This study aimed to mechanically verify if the TrueBeam 4.1 Linac is capable of delivering RAD plans, which introduces a rotating dynamic collimator as a new degree of freedom. To achieve this, two new tests were carried out over a period of 12 weeks. The in‐house developed picket fence test verified the synchronous working of a rotating collimator, gantry, and MLC movement. The Pac‐Man test (designed by Varian) verified the Linac during varying combinations of collimator speed and dose rate with a rotating gantry. For the picket fence test, the results were consistent with a maximum difference of 0.45 mm (±0.05) and a mean difference of 0.14 mm (±0.01). For the Pac‐Man test, the Diff(x) values ranged between −1.31% and 1.02% for the five regions and the $\mathrm{Dif}f_{\mathrm{Abs}}$ value between 0.42% and 0.55%. For both tests, control limits were calculated based on AAPM TG‐218 and all results fell within these limits. Based on the observed stability over 12 weeks and all results remaining within thresholds set in literature, it can be concluded that the delivery of RAD plans is mechanically feasible. From now on, these tests will be implemented in the routine mechanical QC for the RAD‐updated TrueBeam system in UZ Leuven and will be carried out every 2 months.

## AUTHOR CONTRIBUTIONS

Wouter Crijns, Emma Van Riet, Wout Piot, Bianca Vanstraelen, Karel Aerts, Truus Reynders, and Tom Depuydt contributed to the study design. More specifically, Wouter Crijns, Wout Piot, Bianca Vanstraelen, Karel Aerts, and Truus Reynders brainstormed the required tests to mechanically validate RAD; Bianca Vanstraelen attended RAD workshops organized by Varian; Wouter Crijns designed the picket fence test; Tom Depuydt proposed the quantitative consistency analysis using control limits; and Emma Van Riet and Wouter Crijns proposed the validation of the tests. Data acquisition was carried out by Emma Van Riet, Wout Piot, Karel Aerts, Truus Reynders, and other members of the medical physics team at UZ Leuven. Emma Van Riet developed the Python analysis scripts with input and feedback from Wout Piot, Bianca Vanstraelen, Karel Aerts, Truus Reynders, and Wouter Crijns. Data analysis was carried out by Emma Van Riet and interpreted by Emma Van Riet and Wouter Crijns. The manuscript was written by Emma Van Riet and critically reviewed by Wout Piot, Bianca Vanstraelen, Karel Aerts, Truus Reynders, Tom Depuydt, and Wouter Crijns. All authors have approved the final submitted version and agree to be accountable for the accuracy and integrity of the work.

## CONFLICT OF INTEREST STATEMENT

The authors declare no conflict of interest.

## ETHICS STATEMENT

This study did not involve the use of data from human subjects or animals and therefore did not require ethical approval.

## Data Availability

Authors will share data upon request to the corresponding author.
